# Australia's international health relations in 2003

**DOI:** 10.1186/1743-8462-2-3

**Published:** 2005-02-21

**Authors:** Simon Barraclough

**Affiliations:** 1 School of Public Health, La Trobe University, Australia

## Abstract

A survey for the year 2003 of significant developments in Australia's official international health relations, and their domestic ramifications, is presented. The discussion is set within the broader context of Australian foreign policy. Sources include official documents, media reports and consultations with officers of the Department of Health and Ageing responsible for international linkages.

## 

Australia's health relations with other nations in the field of health constitute an important sub-set of health policy not only because of the intrinsic significance of bi-lateral and multilateral linkages, but also because of their ramifications for health policy at the domestic level.

In broad terms, these health relations encompass a range of interactions with consequences for health, including: membership of global and regional bodies; the negotiation of international agreements; action to counter particular external threats to health; assistance to developing countries; and international trade and investment in health-related goods and services. In 2003 there were continuing developments in all these areas within a wider foreign affairs context overshadowed by official policy concerns about global and regional security, the deployment of the Australian armed forces in various theatres of service, and renewed fears of the human and economic costs of infectious diseases. Balancing these concerns with national defence were renewed efforts to forge bi-lateral trade links in global trade environment characterized by the emergence of trade blocs centred in North America, Europe and Southeast Asia.

Although consultation occurs with states and territories, it is the Australian Government that is constitutionally responsible for conducting Australia's international relations. These responsibilities include appointing representatives to international bodies and organizations, such as the United Nations and its various agencies, including the World Health Organization and assenting to agreements and regulations promulgated by international agencies. The formulation and implementation of policy with direct or indirect international health ramifications is not centralized, but is usually the result of consultations between various relevant government departments and statutory authorities.

An important element of the Australian Government's foreign affairs powers relates to international treaties. While a degree of consultation with state and territory governments and with the public occurs, and the national parliament is able to scrutinize and comment upon international treaties, it is the executive that has the final decision on such agreements.

## WHO and other international agencies concerned with health

In 2003 Australia continued to play a strategically important and respected role in international organizations concerned with health, especially the World Health Organization. At the World Health Assembly, the governing body of the WHO, the Australian delegation supported resolutions concerned with strengthening nursing and midwifery and child and adolescent health. In the wake of SARS, Australia also supported the review of the International Health Regulations and is likely to subscribe to them [1]. The voluntary nature of WHO standards and regulations, which can be accepted or rejected by member states, is well illustrated by the International Health Regulations since Australia and Papua New Guinea declined to accept them when they were last promulgated. Australia should be better placed to influence developments in WHO in the next three years as a result of being nominated for a term on the Executive Board.

The Department of Health and Ageing was closely involved with international comparative health data projects including WHO's World Health Survey and the health systems performance survey of the Organization for Economic Cooperation and Development. Australia's participation in the health mandate of the Commonwealth of Nations was illustrated by the Therapeutic Goods Administration's provision of a secretariat for the Clearing House of Commonwealth Agencies for Chemical Safety. Australia also participated in the meeting of Commonwealth health ministers on the eve of the annual World Health Assembly of WHO in Geneva [2].

## International agreements

In December Australia signed the Framework Convention on Tobacco Control (FCTC) the first multilateral treaty negotiated under the auspices of the World Health Organization. For the first time, nations were invited to implement control measures covering such issues as health warnings, advertising, packaging and labelling, sales, and smuggling. They were also called upon to embrace policy measures designed to counter the global tobacco epidemic [3]. The FCTC provided an impetus to the domestic policies of many countries with limited progress on tobacco control and also allowed for the transnational activities of tobacco corporations to be countered with global policy action. The FCTC has limited potential to further Australian domestic policy, which is in advance of that in most countries. If necessary, the Australian Government could call upon its "external affairs" to assert constitutional primacy over this policy area. However, this is unlikely in the context of close cooperation between various levels of government in Australia in establishing national tobacco control policies. Australian leadership was evident in WHO's formulation of the FCTC, having been nominated by the Western Pacific region as vice-chair of the Bureau for the Negotiating Body.

A reciprocal health care agreement with Norway was signed, further expanding the rights of Australian residents to immediate and necessary treatment in the national health systems of countries with which Australia has reciprocal treaties. These include New Zealand, UK, Italy, Malta, Holland, Sweden, Finland, and the Republic of Ireland. These arrangements are "cost neutral" and do not include costly accounting or administrative procedures. In terms of domestic policy, the continuing "internationalisation" of Medicare (pioneered by the Hawke Labor Party ministry at the time of Medicare's introduction) by the Liberal-National Party Coalition is paradoxical since local citizens are being encouraged to opt out of public hospital treatment through a rebate on private health insurance and penalties for higher income earners who do not insure privately. Whilst these agreements have cemented closer diplomatic ties, their potential benefits to international travellers, especially those subject to punitive insurance premiums or the refusal of insurance due to old age or infirmity, remain inadequately publicized. Treaties are also being negotiated with Denmark and Belgium.

Following years of negotiations and planning, a treaty was signed with New Zealand establishing a single joint therapeutic goods agency. This body, due to commence operations in 2005, will regulate prescription and retail drugs, therapeutic devices and also complementary medicines. It will replace the Australian Therapeutic Goods Administration and its New Zealand counterpart. To a large extent, the two regulatory systems will have been integrated, although there are still areas of disagreement (e.g. policies on the advertising of PBS medicines) which will need to be negotiated. This joint agency creates a model in international health relations which other states could profitably emulate where they share common concerns and have similar health systems. In December 2002 the two countries finalized treaty arrangements establishing both a joint standards code and a joint statutory authority, Food Standards Australia New Zealand [4]. These arrangements parallel bi-lateral developments for the joint regulation of food standards.

These developments have furthered Australian foreign policy concerned with establishing trans-Tasman free trade, commenced some two decades ago with the negotiation of the Closer Economic Relations agreement with New Zealand. The new regulatory arrangements have created a virtual trans-Tasman free market in food (subject to plant and animal quarantine considerations) and therapeutic drugs.

While not having the legal status of a treaty, for some years the Department of Health and Ageing has had memoranda of understanding with its counterparts in China, Indonesia, Thailand and Japan. In 2003 further activities were undertaken under the auspices of these agreements. During the state visit of China's president Hu Jinta, a plan of action was signed between the two health ministries. The Indonesian relationship continued with the inclusion of a health delegation to the Sixth Australia Indonesia Ministerial Forum in Jakarta in March, preceded by two rounds of meetings between officials of the Indonesian and Australian health departments. The Australia-Japan Partnership in Health and Family Services formed the basis for negotiations for joint research on mental health and an international conference on suicide prevention [5]. In a related development, the Department of Foreign Affairs and Trade promoted aged care expertise as an export service through the Australia Japan Conference.

In the course of 2003 Australia finalized free trade agreements (in reality, preferential trade agreements) with Singapore and Thailand and continued negotiations with the USA [6]. From the perspective of the Australian health industry, the agreement with Singapore offered tariff-free trade in pharmaceuticals and other therapeutic goods and the gradual removal of tariffs in the case of Thailand. All countries imposed reservations on free trade in the sensitive areas of health services, although traditional Thai massage exponents will be permitted to operate in Australia. Domestically, these agreements required intersectoral policy collaboration in the interests of health. Policy makers in the Department of Health needed to intensify their understanding of the dynamics of international trade, while those making foreign policy had to consider the health dimensions of ostensibly commercial arrangements.

The free trade agreement with the USA raised controversies about attempts to include the Pharmaceutical Benefits Scheme (PBS) in concessions demanded by US negotiators. These issues have been outlined in the account of developments in the PBS elsewhere in this series of review articles.

## SARS

The emergence and rapid spread of Severe Acute Respiratory Syndrome (SARS) to several countries in East and Southeast Asia and to Canada revived popular atavistic fears of pandemics and damaged the tourism and travel industry, as well as some Australian suppliers of goods and services to Asia. WHO issued a global alert on the disease in March, and the last reported case of international occurred in July. So serious was the threat of SARS to the economies of some countries that a special meeting of health ministers, attended by Australia, was organized by Asia-Pacific Economic Cooperation (APEC) to discuss the situation. A task force was subsequently established by APEC to deal with SARS. An example of the economic costs of the disease was the decision by the Governments of Singapore and Australia to postpone negotiations on a greater share of the Sydney-Los Angeles air route (dominated by QANTAS) for the Singapore carrier, due to uncertainty about demand.

In Australia SARS was declared a quarantinable disease under the Quarantine Act 1908 and policy guidelines for health professionals, airline and border control staff and the general public were developed by the Department of Health and Ageing, which also led an inter-departmental task force to monitor world developments. Until July 2003, when the WHO announced that no country was still considered SARS-affected, international aircraft arriving at Australian airports were required to obtain "SARS-free" clearance, nurses were posted at airports and restrictions on elective surgery were placed on travellers returning from affected countries [7]. During the period of WHO's alert, Australia had reported only five probable, and one laboratory-confirmed, case of SARS.

## Health and foreign aid

Australia's official international development assistance programme is an important foreign policy tool, especially in the Asia-Pacific region. Some $225 m. (of a total of $ 1.8 b.) was allocated to health-related international development assistance in 2003–4 budget of the Australian Agency for International Development (AusAID). However, while Australia's contribution to HIV/AIDS control and its regional advisory role associated with SARS were acknowledged by the Foreign Affairs Minister in his report to Parliament, health assistance received little prominence. Security, good governance and counter-terrorism were emphasized as the focus for the official foreign aid programme. Support for essential services in Papua New Guinea continued as a major imperative [8]. The lower priority of health was further underscored by a decision to no longer appoint designated health advisors to the permanent staff of AusAID. It should be noted, however, that the emergence of SARS served to reinforce health as an important element on the international assistance agenda.

## Global health workforce mobility

The fact that the domestic health workforce is now part of a global market for skilled workers was further demonstrated by continuing efforts to recruit nurses from overseas, the decision of the Australian Health Ministers Conference to sanction dentists from selected Commonwealth countries to work in public clinics. In addition, a scheme to recruit overseas-trained medical practitioners was included in the Australian Government's *Medicare Plus *policy initiatives [9]. It is intended that these doctors will work in rural and remote areas officially designated as having medical workforce shortages and also in positions within Aboriginal Controlled Community Health Services. Yet, metropolitan hospitals are have also become reliant upon overseas-trained doctors for their staffing. This policy, accompanied by the liberalization of immigration arrangements for medical doctors, has represented a *volte face *from previous policies deliberately designed to discourage foreign doctors from immigrating in the belief that controlling the number of doctors would contribute to cost-containments of Medicare. It also continues to raise the ethical danger of Australia contributing to a "brain drain" of medical staff from countries that are themselves short of such expertise. In 2002 the Commonwealth of Nations had agreed to a code of practice for the international recruitment of health workers to help safeguard the interests of developing nations. Australia has endorsed the code.

The Australian Government will need to handle policies associated with the recruitment of overseas-trained health personnel with care due to professional sensitivities and the need for legislation at the state level to regularize the status of some professions.

## Concluding observations

This brief review of Australia's international health relations in 2003 has demonstrated that health must be seen as an integral part of trade and security within the wider foreign policy context. The protection of health in free trade arrangements is important for their domestic legitimacy. It is vital that those involved in health policy are aware of its potential international dimensions, while those responsible for foreign affairs include health in their approach. Official health linkages have served to promote good will in some otherwise difficult relationships, as has been the case with Indonesia. They have also helped to promote a positive international image for Australia.

## Note

The opinions expressed in this article are the sole responsibility of the author.
